# Prognostic value of immunohistochemical staining for H3K27me3 and EZH2 in astrocytoma, IDH-mutant

**DOI:** 10.1007/s11060-024-04897-8

**Published:** 2024-12-05

**Authors:** Shumpei Onishi, Fumiyuki Yamasaki, Vishwa Jeet Amatya, Ushio Yonezawa, Akira Taguchi, Iori Ozono, Novita Ikbar Khairunnisa, Yukari Go, Yukio Takeshima, Nobutaka Horie

**Affiliations:** 1 Department of Neurosurgery, Graduate School of Biomedical and Health Sciences, Hiroshima University, 1-2-3 Kasumi, Minami-ku, Hiroshima City, Hiroshima 734-8551 Japan; 2 Department of Pathology, Graduate School of Biomedical and Health Sciences, Hiroshima University, Hiroshima, Japan; 3 Medical Division Technical Center, Hiroshima University, Hiroshima, Japan

**Keywords:** Astrocytoma, IDH-mutant, Glioma, H3K27me3, EZH2

## Abstract

**Background:**

H3 histone 27 lysine (H3K27) trimethylation (H3K27me3), which is catalyzed by enhancer of zeste homolog 2 (EZH2), regulates gene expression through epigenetic mechanisms. H3K27me3 is used as a diagnostic marker for diffuse midline glioma and as a surrogate marker to distinguish posterior fossa ependymoma A and B. However, the clinical significance of the EZH2–H3K27me3 axis in astrocytoma, IDH-mutant has not been reported, prompting this investigation.

**Methods:**

Thirty-three patients with astrocytoma, IDH-mutant treated at our institute were included in this study. Immunohistochemistry (IHC) targeting H3K27me3, H3K27M, EZH2, EZH inhibitory protein, IDH1-R132H, p53, ATRX, Ki-67, and MTAP was performed. Kaplan–Meier analysis and Cox regression analysis were performed to analyze the correlations of overall survival (OS) and progression-free survival (PFS) with various factors, including age, World Health Organization (WHO) grade, the extent of resection, and immunohistochemical results.

**Results:**

The mean patient age was 40.6 ± 11.0 years. IHC for H3K27me3 was positive in 19 patients and negative in 14 patients. The WHO grade and Ki-67 index were significantly higher in the H3K27me3-positive group (*p* = 0.004 and *p* = 0.024, respectively). OS and PFS were significantly shorter in the H3K27me3-positive group (*p* = 0.002 and *p* = 0.026, respectively). Furthermore, the H3K27me3 and EZH2 double-positive group was associated with a higher WHO grade and higher Ki-67 index (*p* = 0.001 and *p* = 0.024, respectively). In the analysis of patients with WHO grade 2/3, double positivity for H3K27me3 and EZH2 was linked to significantly shorter OS and PFS (*p* = 0.0053 and *p* = 0.0048, respectively).

**Conclusion:**

Positivity for H3K27me3, especially double positivity for H3K27me3 and EZH2, could be a poor prognostic factor for astrocytoma, IDH-mutant. These results suggest the utility of H3K27me3 and EZH2 as candidate markers for estimating the malignancy of astrocytoma, IDH-mutant.

## Introduction

In the World Health Organization (WHO) Classification of Tumors of the Central Nervous System updated in 2021, IDH-mutant diffuse astrocytic tumors were classified as “astrocytoma, IDH-mutant” and graded as 2, 3, or 4 depending on the presence of mitotic activity, nuclear atypia, pleomorphism, necrosis, and microvascular proliferation [1]. Astrocytoma, IDH-mutant with homozygous deletion of CDKN2A and/or CDKN2B (CDKN2A/B-HD) carries a poor prognosis [2–4], and CDKN2A/B-HD tumors are graded as “astrocytoma, IDH-mutant, CNS grade 4,” even in the absence of histological features of necrosis or microvascular proliferation [1]. Although grade 2 or 3 tumors are stratified according to anaplasia or mitotic activity, definitive grading for CDKN2A/B-intact astrocytoma, IDH-mutant remains challenging because of differences in inter-rater consensus [5]. Therefore, effective molecular pathological biomarkers, especially for differentiating grade 2 and grade 3 tumors, are required to evaluate the malignancy of astrocytoma, IDH-mutant.

The involvement of H3 histone 27 lysine (H3K27) trimethylation (H3K27me3) in the development of various cancers has been demonstrated. Polycomb repressive complex 2 (PRC2) consists of the enhancer of zeste homolog 2 (EZH2) and several proteins that act as methyltransferases for H3K27. PRC2 regulates gene expression through epigenetic mechanisms, and H3K27me3 is used as a diagnostic or prognostic marker for several types of malignant tumors of the central nervous system [6]. For example, loss of H3K27me3 is related to posterior fossa ependymoma group A, mainly through the expression of EZH inhibitory protein (EZHIP), and is associated with poor prognosis [7, 8]. Moreover, loss of H3K27me3 is one of the diagnostic criteria for H3K27-altered diffuse midline glioma, as lysine 27 to methionine mutation of histone 3 (H3K27M) or EZHIP inhibits PRC2 and reduces H3K27me3 [9]. In addition, combined analysis of H3K27me3 and EZH2 was beneficial in the prognostic prediction of spinal diffuse midline glioma [10].

Previous cohort studies of diffuse gliomas found that loss of H3K27me3 is frequently observed in oligodendroglioma, IDH-mutant and 1p/19q-codeleted, but H3K27me3 could not completely replace the status of 1p/19q codeletion [11, 12]. In another study, H3K27me3 positivity was considered a poor prognostic marker for diffuse glioma independent of IDH mutation or 1p/19q codeletion, but its prognostic value based on the current WHO classification was not evaluated [13]. As higher-grade diffuse glioma tends to exhibit H3K27me3 expression, immunohistochemistry (IHC) for H3K27me3 could potentially predict the prognosis of astrocytoma, IDH-mutant. To elucidate the clinical value of the EZH2–H3K27me3 axis in astrocytoma, IDH-mutant, we analyzed the pathological and clinical characteristics of this entity.

## Methods

### Patients

This retrospective study was approved by the Ethical Committee for Epidemiology of Hiroshima University (E2022-0038). Because of the retrospective nature of the study, the Ethical Committee for Epidemiology of Hiroshima University waived the need for informed consent. All methods were performed in accordance with the relevant guidelines and regulations. We included 33 patients with newly diagnosed astrocytoma, IDH-mutant treated at our institute between January 2009 and May 2024. Patients with insufficient tissue samples for pathological examinations were excluded from this study.

### Histopathological diagnosis and molecular signature analysis

Surgically resected tumor specimens were fixed in 10% phosphate-buffered formalin and embedded in paraffin blocks. Representative slides were then stained with hematoxylin-eosin (HE) for standard histological diagnosis as described previously [14]. IHC was performed with automated immunostainers (BenchMark GX; Ventana, Tucson, AZ, USA) using the ultraView Universal DAB detection kit (Roche Diagnostics International AG, Rotkreuz, Switzerland). The primary antibodies were rabbit monoclonal anti-H3K27me3 (1:200, #9733, Cell Signaling Technology, Danvers, MA, USA), rabbit monoclonal anti-EZH2 (1:100, 790–4651, Cell Signaling Technology), anti-H3K27M (#ABE419, Millipore, Billerica, MA, USA; 1 µg/mL), rabbit monoclonal anti-CXorf67 antibody (for EZHIP; 1:100, polyclonal; Sigma-Aldrich, St. Louis, MO, USA), mouse monoclonal anti-human IDH1-R132H (DIA-H09, Dianova, Hamburg, Germany; 20 µg/mL), rabbit polyclonal anti-ATRX (HPA001906, Sigma-Aldrich; 5 µg/mL); monoclonal mouse anti-human p53 protein (NCL-L-p53-DO7, Leica, Nussloch, Germany; 10 µg/mL), and rabbit monoclonal anti-human Ki-67 (30 − 9, Roche, Basel, Switzerland; 2 µg/mL).

According to previous research [11, 13], H3K27me3 immunohistochemical expression was classified as “positive” when nuclear staining was observed in ≥ 5% of neoplastic cells or as “negative” when staining was absent in > 95% of neoplastic cells and present in internal positive controls (endothelium, neurons). Positive EZH2 staining was indicated by strong nuclear staining [15]. The cutoff for ATRX and p53 positivity was 10% nuclear staining as previously described [14]. The labeling index evaluation for Ki-67 was based on nuclear staining in tumor cells.

Tumors were diagnosed according to the WHO classification for central nervous system tumors updated in 2021 through consensus by two authors (V.J.A. and Y.T.). In some cases, tumor molecular profiling including CDKN2A/B was confirmed by a next-generation sequencing-based comprehensive genomic profiling test using the FoundationOne^®^CDx (Foundation Medicine, Cambridge, MA, USA) or Oncomine™ Childhood Cancer Research Assay (Thermo Fisher Scientific, Waltham, MA, USA) as previously described [16].

### Magnetic resonance imaging (MRI) and computed tomography (CT) acquisition and evaluation

MRI was performed using 3.0T scanners (Ingenia CX 3.0T; Philips Healthcare, Best, Netherlands, or Signa Excite HD 3.0T; GE Healthcare, Chicago, IL, USA). Investigators evaluated the T1-weighted imaging (T1WI), T2-weighted imaging (T2WI), T2*-weighted imaging, fluid-attenuated inversion recovery (FLAIR), diffusion-weighted imaging, and postcontrast T1WI sequences of each patient. The MRI parameters were used as previously described [17, 18]. Contrast enhancement was classified as positive if part of the tumors was enhanced regardless of the enhanced proportion. T2-FLAIR mismatch was defined similarly to the original description of the sign. The sign describes a homogeneously hyperintense signal on T2WI and peripheral hyperintensity with central hypointensity on FLAIR imaging [19–21]. If the tumor displayed contrast enhancement, then it was classified as T2-FLAIR mismatch sign-negative.

CT was performed on a 320-detector CT scanner (Aquilion ONE; Canon Medical Systems, Ōtawara, Japan) or a 16-slice helical CT scanner (LightSpeed Ultra, GE Healthcare). Intratumoral calcification was evaluated using the CT findings.

### Statistical analysis

Fisher’s exact test was used to compare patient characteristics between the groups and assess the association of the H3K27me3 status with molecular pathological and clinical characteristics. The Mann–Whitney *U* test was used to compare age and the Ki-67 index. Kaplan–Meier analysis and Cox regression analysis were performed to analyze overall survival (OS) and progression-free survival (PFS) in relation to various factors, including age, WHO grade, the extent of resection, and immunohistochemical results. The level of significance was set at *p* < 0.05. JMP Pro 17.0 (SAS, Cary, NC, USA) and GraphPad Prism, ver. 10.3.1 for Mac (GraphPad Software, Boston, MA, USA) were used for statistical analyses.

## Results

### Clinical and molecular pathological features

This study included 33 patients (12 women and 21 men; median age, 41 years; range, 19–68 years) with astrocytoma, IDH-mutant. According to the 2021 WHO classification, the tumors were classified as “astrocytoma, IDH-mutant, grade 2;” “astrocytoma, IDH-mutant, grade 3;” and “astrocytoma, IDH-mutant, grade 4” in 21, 3, and 9 patients, respectively. Of the nine cases of grade 4 astrocytoma, IDH-mutant, four were classified based on pathological features (microvascular proliferation and necrosis), four were classified based on molecular features (CDKN2A/B HD), and one was classified based on both pathological and molecular feature. All tumors were positive on IHC for anti-IDH1-R132H and negative for anti-H3K27M and anti-EZHIP. Only four tumors retained positivity for anti-ATRX, and the diagnosis of astrocytoma in these cases was confirmed by 1p/19q non-codeletion via fluorescence in situ hybridization.

Subsequently, the patients were divided into two groups according to H3K27me3 expression on IHC. The WHO grade (*p* = 0.0036, Fisher’s exact test) and Ki-67 index (*p* = 0.0241, Mann–Whitney *U* test) were significantly higher in the H3K27me3-positive group. CDKN2A/B HD or MTAP loss showed a higher number in the H3K27me3-positive group, but they did not show statistical significance. Among only astrocytoma, grade 4, these factors did not show an association. Temozolomide was used more frequently in the H3K27me3-positive group (*p* = 0.024). No differences were recorded between the groups for age, sex, the extent of resection, and radiation therapy. Concerning the MRI characteristics, contrast enhancement was observed more frequently in the H3K27me3-positive group (*p* = 0.027, Fisher’s exact test). The incidence of the T2-FLAIR mismatch sign did not differ between these groups. Details of the patients are summarized in Table 1.

**Table 1:** Characteristics of patients with astrocytoma, IDH-mutant according to the status of H3K27me3 expression

	H3K27me3- positive astrocytoma	H3K27me3-negative astrocytoma	*p*
N	19	14	
Age, years	40.1 ± 11.4	41.14 ± 10.8	0.7522
Sex (male/female)	11/8	10/4	0.4861
WHO grade			
Grade 2	8	13	0.0036
Grade 3	3	0	0.2443
Grade 4	8	1	0.0466
Necrosis or microvascular proliferation	4	1	0.3662
CDKN2A/B HD or MTAP loss	4	1	0.3662
Ki-67 index, median (range)	8% (1–90)	3% (1–30)	0.0241
Location			
Frontal	15	9	0.4421
Temporal	2	1	1.0000
Insular	1	3	0.2882
Others	1	1	1.0000
Radiological characteristics			
Contrast enhancement	6	0	0.0272
T2-FLAIR mismatch	6	8	0.1728
Calcification	2	5	0.1061
Treatment			
Surgical resection			
Total	5	6	0.2728
Non-total	14	8	
Temozolomide	16	6	0.0240
Radiation therapy	16	9	0.2390

*p < 0.05

**Fig. 1:**
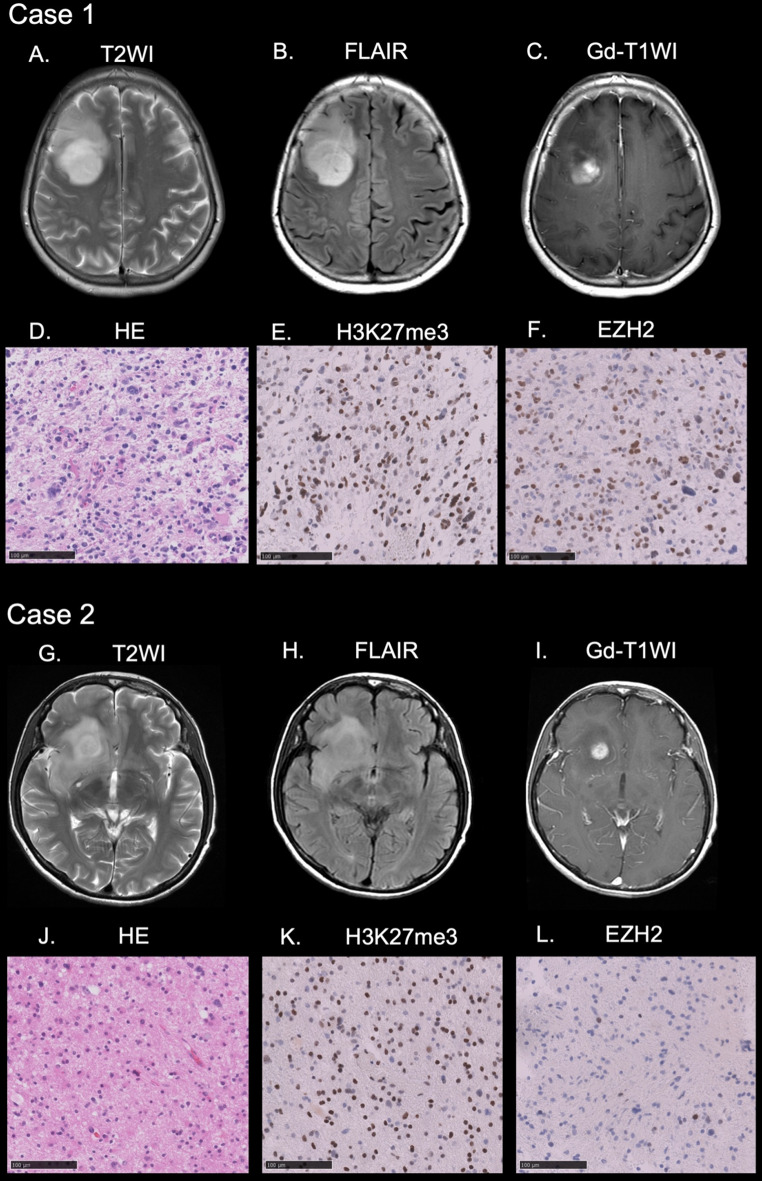
Case 1. MRI revealed a right frontal lobe tumor with hyperintensity on T2WI (**A**) and FLAIR (**B**). The tumor was partially enhancing on post-contrast T1WI (**C**). HE revealed a diffuse astrocytic tumor (**D**). IHC revealed positivity for H3K27me3 (**E**) and EZH2 (**F**). Case 2. MRI revealed a right frontal lobe and insular tumor with hyperintensity on T2WI (**G**) and FLAIR (**H**). The tumor was partially enhancing on post-contrast T1WI (**I**). HE revealed a diffuse astrocytic tumor (**J**). IHC uncovered positivity for H3K27me3 (**K**) and negativity for EZH2 (**L**)

**Fig. 2:**
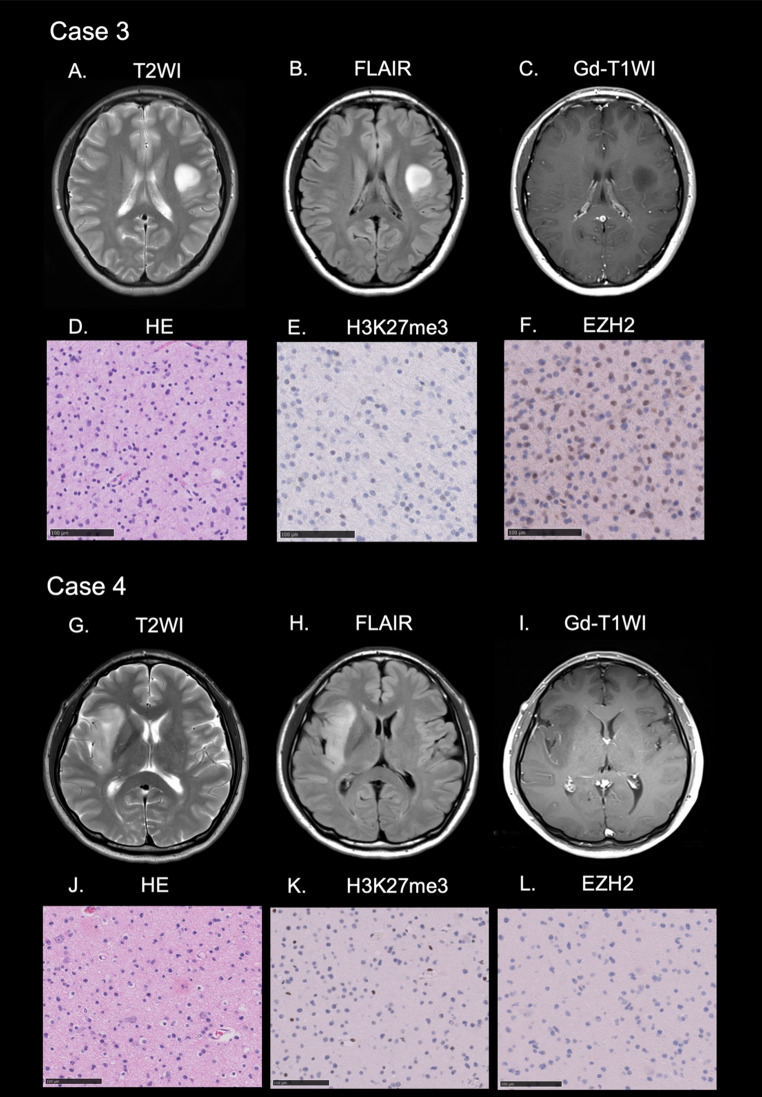
Case 3. MRI revealed a left frontal tumor with hyperintensity on T2WI (**A**) and FLAIR (**B**). The tumor displayed no contrast enhancement on post-gadolinium T1WI (**C**). HE revealed a diffuse astrocytic tumor (**D**). IHC disclosed the loss of H3K27me3 (**E**) and positivity for EZH2 (**F**). Case 4. MRI revealed a right frontal lobe and insular tumor with hyperintensity on T2WI (**G**) and FLAIR (**H**). The tumor displayed no contrast enhancement (**I**). HE revealed a diffuse astrocytic tumor (**J**). IHC disclosed the loss of H3K27me3 (**K**) and loss of EZH2 (**L**)

Representative cases of astrocytoma, IDH-mutant are presented in Figs. 1 and 2.

### The relationship of the H3K27me3 status with prognosis

**Fig. 3:**
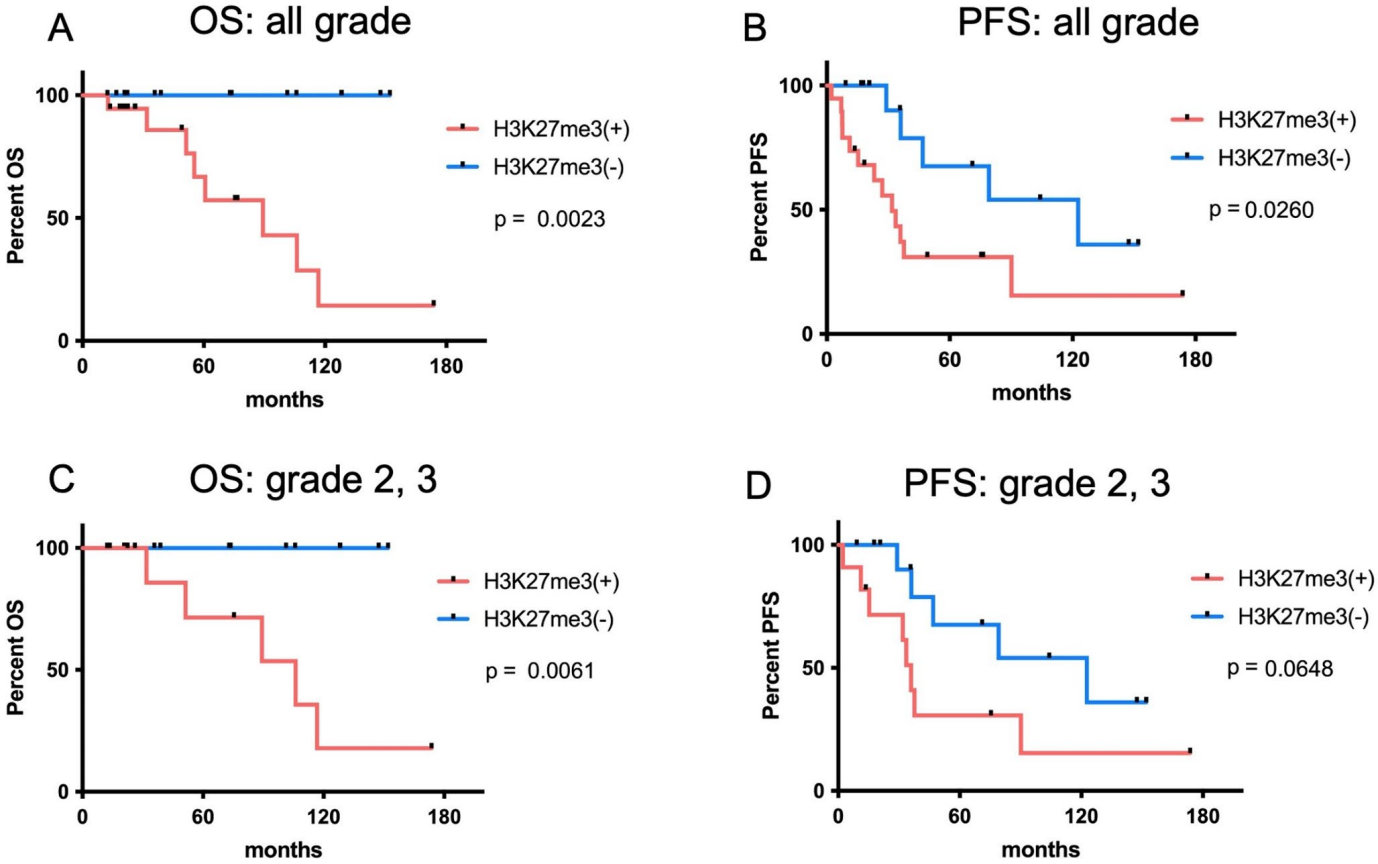
Kaplan–Meier analysis of OS and PFS in patients with astrocytoma, IDH-mutant according to H3K27me3 expression. OS (**A**) and PFS (**B**) among all WHO grades and OS (**C**) and PFS (**D**) among WHO grades 2 and 3 are presented

IHC for H3K27me3 was positive in 19 patients and negative in 14 patients. The H3K27me3-positive group exhibited significantly shorter OS and PFS than the H3K27me3-negative group (*p* = 0.0023 and *p* = 0.0260, respectively, log-rank test; Fig. 3A, B). The median OS was 89.3 months in the H3K27me3-positive group and not reached in the H3K27me3-negative group. The median PFS was 31.9 months in the H3K27me3-positive group and 122.7 months in the H3K27me3-negative group. Among the patients with WHO grade 2/3 tumors, OS was significantly shorter in those with H3K27me3 positivity than in those with H3K27me3 negativity (*p* = 0.0061; Fig. 3C), the median OS was 106.1 months in the H3K27me3-positive group and was not reached in the H3K27me3-negative group. The H3K27me3-positive patients tended to have shorter PFS (*p* = 0.0648; Fig. 3D), the median PFS was 35.9 months in the H3K27me3-positive group and 122.7 months in the H3K27me3-negative group.

### The relationships of the H3K27me3 and EZH2 statuses with prognosis

Multivariate Cox regression analysis for OS was performed using H3K27me3, EZH2, WHO grade, the extent of resection, the Ki-67 index, and contrast enhancement on MRI as the variables. H3K27me3 was most strongly predictive of shorter OS (*p* = 0.0349), followed by EZH2 (*p* = 0.051).

Accordingly, the patients were subclassified according to H3K27me3 and EZH2 expression. The WHO grade (*p* = 0.0091, Fisher’s exact test) and Ki-67 index (*p* = 0.0241, Mann–Whitney *U* test) were significantly higher in the H3K27me3/EZH2 double-positive group. CDKN2A/B HD or MTAP loss showed a higher number in the H3K27me3/EZH2 double-positive group, but they did not show statistical significance. There were no significant differences between the groups concerning age, sex, the extent of resection, use of temozolomide, and radiation therapy. Regarding the MRI characteristics, the contrast enhancement showed high specificity for the double-positive group (35.7% sensitivity, 94.7% specificity, AUC = 0.652) and was observed more frequently in the H3K27me3/EZH2 double-positive group, but they did not show the statistical difference (*p* = 0.0616, Fisher’s exact test). The details of the patient’s characteristics are summarized in Table 2.

**Table 2:** Characteristics of patients with astrocytoma, IDH-mutant according to the double positivity for H3K27me3 and EZH2

	H3K27me3- and EZH2-positive astrocytoma	The other astrocytoma	*p*
N	14	19	
Age, years	39.7 ± 12.5	41.3 ± 10.1	0.5584
Sex (male/female)	8/6	13/6	0.7157
WHO grade			
Grade 2	5	16	0.0091
Grade 3	2	1	0.5612
Grade 4	7	2	0.0191
Necrosis or Microvascular proliferation	3	2	0.6285
CDKN2A/B HD or MTAP loss	4	1	0.1376
Ki-67 index, median (range)	11.3% (1–90)	2.8% (1–40)	0.0241
Location			
Frontal	11	13	0.6982
Temporal	1	2	1.0000
Insular	1	3	0.6197
Others	1	1	1.0000
Radiological characteristics			
Contrast enhancement	5	1	0.0616
T2-FLAIR mismatch	4	10	0.2857
Calcification	1	6	0.1950
Treatment			
Surgical resection			
Total	4	8	0.4861
Non-total	10	11	
Temozolomide	12	10	0.0674
Radiation therapy	11	13	0.4157

*p < 0.05

**Fig. 4:**
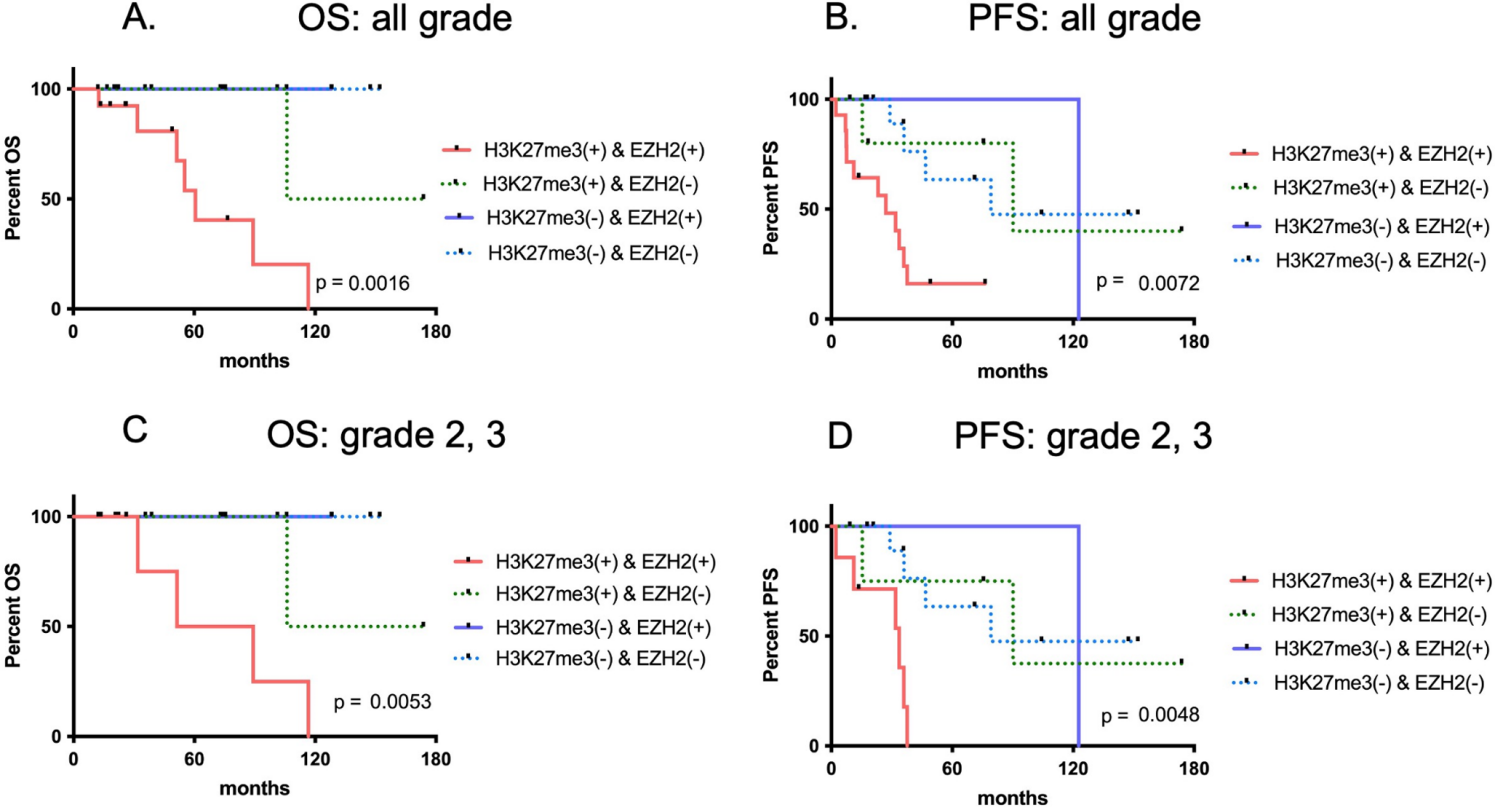
Kaplan–Meier analysis of OS and PFS in patients with astrocytoma, IDH-mutant according to H3K27me3 and EZH2 expression. OS (**A**) and PFS (**B**) among all WHO grades and OS (**C**) and PFS (**D**) among WHO grades 2 and 3 are presented

Patients with positivity for both H3K27me3 and EZH2 exhibited significantly shorter OS and PFS than those without double positivity (*p* = 0.0016 and *p* = 0.0072, respectively; Fig. 4A, B). The median OS was as follows: 60.7 months in H3K27me3/ EZH2 double-positive group, 140.0 months in the H3K27me3 positive and EZH2-negative group, not reached in the H3K27me3-negative and EZH2-positive group and in H3K27me3/ EZH2 double negative group. Among H3K27me3-negative patients, the survival curves for EZH2-positive and EZH2-negative patients overlapped (Fig. 4A). The median PFS was as follows: 27.0 months in the H3K27me3/EZH2 double-positive group, 90.1 months in the H3K27me3-positive and EZH2 negative-group, 122.7 months in the H3K27me3-negative and EZH2-positive group, and 79.1 months in the H3K27me3/EZH2 double-negative group.

Next, survival analyses were performed only among patients with WHO grade 2 or 3 tumors. OS and PFS were significantly shorter in patients positive for both H3K27me3 and EZH2 (*p* = 0.0053 and *p* = 0.0048, respectively; Fig. 4C, D). The median OS was as follows: 70.3 months in the H3K27me3/EZH2 double-positive group, 140.0 months in the H3K27me3-positive and EZH2-negative group, not reached in the H3K27me3-negative and EZH2-positive group and the H3K27me3/EZH2 double-negative group. Kaplan–Meier analysis also revealed an overlap of the survival curves for the EZH2-positive and EZH2-negative subgroups of H3K27me3-negative patients (Fig. 4C). The median PFS was as follows: 33.6 months in the H3K27me3/EZH2 double-positive group, 90.1 months in the H3K27me3-positive and EZH2-negative group, 122.7 months in the H3K27me3-negative and EZH2-positive group, and 79.1 months in the H3K27me3/EZH2 double-negative group.

## Discussion

Our study revealed that H3K27me3 expression was associated with poor prognosis in astrocytoma, IDH-mutant. In addition, the combination of the H3K27me3 status and EZH2 expression could be a novel prognostic marker for this malignancy.

Although the clinical significance of H3K27me3 has been reported in various cancers, the clinical interpretation of H3K27me3 varies among different tumors. Loss of H3K27me3 was linked to poor prognosis or treatment resistance in posterior fossa ependymoma [7], medulloblastoma [22], breast cancer [23], meningioma [24], acute lymphoblastic lymphoma [25], chordoma [26], colon cancer [27], and non-small cell lung cancer [28]. In general, H3K27me3 represses gene transcription and regulates proliferation and differentiation in the growth process. As one of the mechanisms of treatment resistance, loss of H3K27me3 results in increased expression of EPHA2, a tyrosine kinase that stimulates the AKT signaling pathway in medulloblastoma [22]. By contrast, H3K27me3 expression has the opposite effect in certain tumors. H3K27me3 positivity was associated with poor prognosis in esophageal cancer [29], melanoma [30], hepatocellular cancer [31], gastric cancer [32], and urothelial cancer [33]. Even among diffuse glioma, astrocytoma and diffuse midline glioma show opposite expression patterns of H3K27me3 [34]. In diffuse midline glioma, H3K27M binds and inhibits PRC2, which induces the reduction of H3K27 methylation [35]. And H3K27M tumor cells use alpha-ketoglutarate (a-KG) to maintain low global H3K27me3 levels [34]. On the other hand, IDH1/2 mutations metabolize a-KG to D-2-hydroxyglutarate (D2HG) [36]. Excessive D2HG inhibits the catalytic efficiency of the Histone demethylases (KDMs), which increase the H3K27me3 level [34, 37]. Our study focusing on astrocytoma, IDH-mutant illustrated that H3K27me3 expression was associated with poor prognosis. A previous study revealed a shift in the enrichment site of H3K27me3 modification in diffuse glioma [38] and suggested that this shift alters the profile and plays an important role in tumorigenesis and progression.

Furthermore, to reveal the mechanisms of H3K27me3 expression in astrocytoma, IDH-mutant, we also evaluated EZH2. EZH2 has paradoxical activity, serving as both a transcription suppressor and oncogene. EZH2, the catalytic subunit of PRC2, acts primarily as a gene transcription silencer via trimethylation of H3K27. EZH2 also has a PRC2- and H3K27me3-independent role in cancer [39]. For instance, EZH2 enhances STAT3 activity by increasing STAT3 tyrosine phosphorylation in glioblastoma [40]. In addition, EZH2 overexpression increases NOTCH1 expression and signaling. These oncogenic activities of EZH2 might be related to the malignancy in EZH2-positive astrocytoma. In a previous study, high-grade astrocytic glioma exhibited higher EZH2 expression than low-grade glioma or normal brain tissue [38]. Furthermore, diffuse midline glioma with high EZH2 expression carried a worse prognosis than diffuse glioma and diffuse midline glioma with low EZH2 expression [15, 39].

Analyzing the co-expression of H3K27me3 and EZH2 could be a feasible approach for predicting prognosis. Our study found that the H3K27me3 negative astrocytoma patients showed poor prognosis. The IHC of EZH2 could stratify the prognosis of H3K27me3 negative patients and co-expression of H3K27me3 and EZH2 showed poor prognosis compared with H3K27me3-positive and EZH2-negative group. In the paired analysis of astrocytoma, IDH-mutant, increased H3K27me3 and EZH2 expression was observed during progression [41], and these alternations could be associated with the malignant progression of astrocytoma, IDH-mutant. Furthermore, in a genome-wide ChIP-seq analysis of EZH2-mediated H3K27me3, high-grade glioma displayed a higher rate of H3K27me3 gene modification than low-grade glioma, and the extent of medication was similar to that of embryonic stem cells [38]. This embryonic stem cell-like methylation profile might be associated with treatment resistance or poor prognosis.

Some limitations of our study should be acknowledged. This retrospective study involved a small number of patients with astrocytoma, IDH-mutant. In particular, the H3K27me3-negative and EZH2-positive group consisted of only two patients in this study. Although the number of cases is small due to the single-center retrospective study, this novel finding is considered highly significant. Further research based on the findings is necessary to validate the efficacy of H3K27me3/EZH2 for predicting the malignancy of astrocytoma, IDH-mutant and to explore the association of H3K27me3/EZH2 with the molecular features of CDKN2A/B HD or the radiological characteristics.

## Conclusion

Positive H3K27me3 immunostaining was a poor prognostic factor in astrocytoma, IDH-mutant. Moreover, double positivity for H3K27me3 and EZH2 could portend a poor prognosis for this tumor.

## Data Availability

No datasets were generated or analysed during the current study.
